# Isocitrate Dehydrogenase Mutations in Glioma: Genetics, Biochemistry, and Clinical Indications

**DOI:** 10.3390/biomedicines8090294

**Published:** 2020-08-20

**Authors:** Yang Liu, Fengchao Lang, Fu-Ju Chou, Kareem A. Zaghloul, Chunzhang Yang

**Affiliations:** 1 Neuro-Oncology Branch, Center for Cancer Research, National Cancer Institute, Bethesda, MD 20892, USA; yang.liu5@nih.gov (Y.L.); fengchao.lang@nih.gov (F.L.); fu-ju.chou@nih.gov (F.-J.C.); 2 Surgical Neurology Branch, National Institute of Neurological Disorders and Stroke, National Institutes of Health, Bethesda, MD 20892, USA; kareem.zaghloul@nih.gov

**Keywords:** *IDH* mutation, glioma, cancer, therapy resistance

## Abstract

Mutations in isocitrate dehydrogenase (*IDH*) are commonly observed in lower-grade glioma and secondary glioblastomas. *IDH* mutants confer a neomorphic enzyme activity that converts α-ketoglutarate to an oncometabolite D-2-hydroxyglutarate, which impacts cellular epigenetics and metabolism. *IDH* mutation establishes distinctive patterns in metabolism, cancer biology, and the therapeutic sensitivity of glioma. Thus, a deeper understanding of the roles of *IDH* mutations is of great value to improve the therapeutic efficacy of glioma and other malignancies that share similar genetic characteristics. In this review, we focused on the genetics, biochemistry, and clinical impacts of *IDH* mutations in glioma.

## 1. Introduction

In 2008, compelling research showed that mutations in isocitrate dehydrogenase (*IDH1* and *IDH2*) are frequently identified in the World Health Organization (WHO) grade II/III gliomas and secondary glioblastomas (GBMs). In contrast, these mutations are rare in primary GBM patients [1]. In 2009, Yan et al. [2] showed that *IDH1* and *IDH2* mutations frequently occur in WHO grade II/III astrocytomas and oligodendrogliomas. Besides gliomas, *IDH* mutations also occur in other non-central nervous system (CNS) malignancies, including acute myeloid leukemia (AML) [3,4], intrahepatic cholangiocarcinoma [5,6], chondrosarcoma [7], and melanoma [8,9]. The mutations are confined to a single arginine residue (Arg^132^) in *IDH1* or two arginine residues (Arg^172^ and Arg^140^) in *IDH2* [10,11]. The mutations commonly cause amino acid substitutions, which localize at the active sites of the enzymes and alter the catalytic functions of *IDH* enzymes. In contrast to wild-type *IDH*, which transforms isocitrate into α-ketoglutarate (α-KG), the mutated *IDH*s convert α-KG into D-2-hydroxyglutarate (D-2-HG) [12]. The altered catalytic activity that occurs because of cancer-associated *IDH* mutations was later termed “neomorphic activity”. The overproduction of the oncometabolite D-2-HG leads to widespread physiological consequences, including profound effects on cellular metabolism [13,14], epigenetic shift [15,16,17,18], genomic instability [19,20,21,22,23], and redox homeostasis [24,25,26,27,28,29]. *IDH* mutations are considered founder events for oncogenesis, through which an ancestor glial cell commits to malignant transformation. On the other hand, the mutant *IDH* enzyme brings about substantial changes in cancer biology, thereby establishing novel therapeutic vulnerabilities that are not commonly identified in other neoplasms. In the present review, we provide an overview of the current knowledge regarding *IDH* mutations in glioma and discuss the distinctive features in terms of genetic, biochemical, and clinical indications in detail.

## 2. Genetics of Glioma

### 2.1. The Prevalence of IDH Mutation in Glioma (Lower-Grade Glioma and GBM)

**Figure 1:**
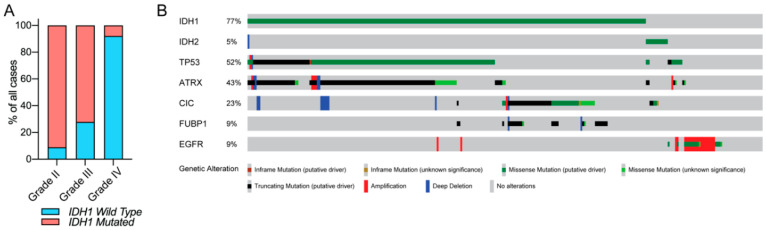
Isocitrate dehydrogenase (*IDH*) mutation in glioma. (**A**) The prevalence of the *IDH1* mutation in glioma based on the pathologic grade. (**B**) Common genetic alterations in lower-grade glioma (LGG). *IDH1*/*2* missense mutations are frequently observed in LGG. *IDH*-mutated LGG frequently harbors missense or truncating mutations in *TP53*, *ATRX*, capicua transcriptional repressor (*CIC*), and far upstream element binding protein 1 (*FUBP1*). *EGFR* gene amplifications and/or missense mutations are observed frequently in *IDH* wild-type gliomas, but rarely so in *IDH*-mutated cases. The data are visualized through cBioPortal [46,47].

*IDH* mutations occur in approximately 80% of all WHO grade II/III gliomas (also known as lower-grade glioma (LGG)) and secondary GBMs [2,30]. In contrast, *IDH* mutations only account for less than 5% of clinical cases of GBM, suggesting that LGG and GBM are minimally overlapping disease subtypes (Figure 1A). The aggregate data from many preclinical and clinical studies have shown that *IDH* mutations alone are insufficient for malignant transformation [31,32]. *IDH* mutations occur at the early stage of gliomagenesis, and often acquire secondary genetic abnormalities such as mutations in tumor protein 53 (*TP53*), loss of ATP-dependent helicase ATRX, X-linked helicase II (*ATRX*), or chromosomal region 1p/19q co-deletion. These alterations correlate to the histological classification of the disease. For example, *IDH* mutant diffuse astrocytomas frequently harbor *TP53* mutations and loss of *ATRX* [33,34,35], while these changes are less likely to be observed in most *IDH* mutant oligodendrogliomas [35,36]. In contrast, most histologically confirmed *IDH* mutant oligodendrogliomas harbor 1p/19q co-deletion [37,38,39,40,41]. Interestingly, a majority of glioma patients with *IDH* mutation and 1p/19q co-deletion also acquire mutations in the promoter regions of the telomerase reverse transcriptase (*TERT*) [36,42]. Moreover, mutations of homolog of *Drosophila* capicua transcriptional repressor (*CIC*) and far upstream element binding protein 1 (*FUBP1*) occur frequently in tumors with 1p/19q loss in oligodendrogliomas [37,41]. In contrast, these secondary genetic abnormalities are rare in *IDH* wild-type gliomas, while *EGFR* amplification appears to occur more frequently compared with *IDH* mutant gliomas (Figure 1B) [1,43]. Recently, an exome sequencing screening in *EGFR*-mutated gliomas identified multiple cooperating partner genes that are associated with *EGFR* driving gliomagenesis, including *Cdkn2a*, *Nf1*, *Spred1,* and *Nav3*. However, *IDH* mutations are not associated with *EGFR*, suggesting no cooperation between these two genes during gliomagenesis [44]. A combination of these genetic signatures and histology provides extra information for accurate glioma subtype classification, and aids in differential diagnosis. With the high prevalence and distinct clinical phenotype of *IDH*-mutated glioma, the WHO updated the classification of CNS malignancies, with an emphasis on the consideration of *IDH* mutations as a marker for genetic diagnosis [45].

### 2.2. Hypermethylation Phenotype in a Subset of IDH-Mutated Gliomas

Several clinical studies have shown that *IDH* mutations correlate significantly with both global DNA hypermethylation and histone methylation [18,48,49]. Through an analysis of a dataset from The Cancer Genome Atlas (TCGA) containing 272 GBM cases, Noushmehr et al. [48] reported that a subset of gliomas exhibits extensive DNA methylation throughout the genome. This was later termed glioma-CpG Island Methylator Phenotype (G-CIMP). Furthermore, the researchers validated G-CIMP in non-TCGA GBMs and LGGs, and revealed a strong association between G-CIMP and *IDH1* mutations. A similar study conducted by Christensen et al. [49] showed vastly different methylation patterns across glioma tumor histological subtypes. The *IDH* mutations were strongly associated with a substantial increase in hypermethylation loci, despite their histological diversity. Through in vitro studies, Turcan et al. [18,50] demonstrated that the acquisition of *IDH* mutation is sufficient to establish the genome-wide hypermethylation of CpG islands, which recapitulate the G-CIMP in patients. These findings indicate that *IDH* mutations play a critical role in epigenetic modulation in gliomas. The CpG island methylator phenotype could be lost through the progression of *IDH*-mutated glioma, which mimics *IDH* wild-type stem cell-like GBM, and generally predisposes patients to a poor clinical outcome [51].

## 3. Biochemistry of *IDH*

### 3.1. Wild-Type IDH

In 1939, Adler et al. [52] first isolated *IDH* from animal tissue. They demonstrated that this enzyme catalyzes the transformation of isocitrate to α-keto-β-carboxyglutaric acid, which spontaneously yields α-ketoglutaric acid and CO_2_.

$$Isocitrate+NAD{\left(P\right)}^{+}⇌\alpha -KG+CO_{2}+NAD(P)H+H^{+}$$

**Figure 2:**
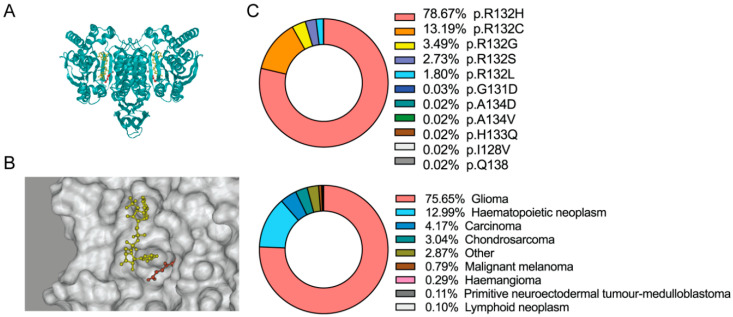
Cancer-associated-*IDH1* mutation. (**A**) Ribbon diagrams of the *IDH1* structure in *Homo sapiens*. NADP is highlighted in yellow. α-Ketoglutarate (α-KG) is highlighted in red. The structure is visualized based on the known crystallography 4KZO [55]. (**B**) The structure of the catalytic center of the *IDH1* enzyme. NADPH is highlighted in yellow. α-KG is highlighted in red. The structure is visualized based on the known crystallography 4KZO. (**C**). The frequency of *IDH1* somatic mutations (upper panel) and *IDH1* mutations in human malignancies (lower panel). Percentages were calculated from the Catalogue of Somatic Mutations in Cancer (COSMIC) database [56].

Since *IDH* was discovered, investigations through the decades have revealed that it plays an essential role in numerous pivotal biological processes, including the Krebs cycle, glutamine metabolism, lipid synthesis, and redox homeostasis [53]. Human cells express three isoforms of *IDH*. *IDH1* is localized in the cytoplasm and peroxisomes, whereas *IDH2* and *IDH3* are found in the mitochondrial matrix. Despite the differences in their localization, *IDH1* and *IDH2* are both heterodimers and exhibit a high similarity in gene sequences (Figure 2A). *IDH1* and *IDH2* catalyze identical, reversible reactions, as follows: decarboxylation of isocitrate to form α-KG, while reducing NADP^+^, as a cofactor, to form NADPH (Figure 2B). *IDH3* is a holoenzyme that comprises two α_2_βγ heterotetramers. Although *IDH3* comprises a complex with more subunits, it catalyzes a similar reaction to decarboxylate isocitrate, and uses NAD^+^ as a cofactor [54]. As cancer-associated mutations occur predominantly in *IDH1*, we mainly focus on *IDH1* mutants and their impact on cancer biology.

### 3.2. IDH Neomorphic Activity and D-2-HG

Cancer-associated *IDH* mutations are mostly missense mutations, which lead to amino acid substitutions at specific arginine residues in the core active sites. In *IDH1*, the amino acid substitution commonly affects Arg^132^, whereas in *IDH2*, the mutations cluster in Arg^140^ or Arg^172^. The R132H and R132C variants of the *IDH1* mutations are the most observed somatic changes in human malignancies, and are identified in 91.86% of all *IDH1*-mutated cancers (Figure 2C). The *IDH1* R132H variant is predominantly found in gliomas, hematopoietic cancers, carcinoma, and chondrosarcoma (Figure 2C). The amino acid substitution from Arg^172^ to lysine (K) is the most common variant present in *IDH2*-mutated glioma [40]. *IDH2* Arg^132^ and *IDH2* Arg^172^ are highly conserved in the catalytic active sites, and play critical roles in the recognition of their substrate isocitrate [57]. It has been reported that *IDH* mutations lead to impaired NADPH production and a decreased affinity for isocitrate, which may suggest that mutations yield a dominant negative inhibition of the enzymes [2]. However, several subsequent investigations, particularly the pioneering work by Dang et al. [58], have reported that cancer-associated *IDH1* mutations led to an alteration in the substrate preference of the enzyme, such that the mutant enzyme exhibits a higher affinity for α-KG. In addition, *IDH1* mutation results in a neomorphic activity that converts α-KG to D-2-HG, in an NADPH-dependent manner. Similarly, *IDH2* mutants also showed a neomorphic activity by producing D-2-HG in glioma and leukemia [11,59].

$$\alpha -KG+NAD(P)H\to D-2-HG+NAD{\left(P\right)}^{+}$$

### 3.3. Distinctive Biological Patterns of IDH-Mutated Glioma

The neomorphic activity of *IDH* mutants results in the production of a large quantity of D-2-HG. The accumulation of D-2-HG impacts cancer biology by affecting α-KG-dependent enzymes, which establishes a distinctive phenotype in the *IDH*-mutated glioma [15,16]. D-2-HG influences a wide spectrum of molecular pathways, including those of epigenetic modulation, DNA repair, metabolism, redox balance, and the immune system. Investigations on these pathways provide a deep understanding of the *IDH* mutations in glioma biology, and justifications for targeting these pathways in the treatment of *IDH* mutant gliomas.

### 3.4. Histones and DNA Demethylases

The chemical structure of D-2-HG (α-hydroxyglutaric acid) is similar to that of α-KG (2-oxoglutaric acid). The accumulation of D-2-HG likely affects the oxidoreductases that use α-KG as a cofactor. D-2-HG has been shown to inhibit multiple histone demethylases, including KDM7A (demethylate H3K9me2 and H3K27me2) [15], KDM4A/B (demethylate H3K9) [60], and H3K36 demethylases [61]. The pioneering research conducted by Xu et al. [15] demonstrated that D-2-HG competitively inhibits α-KG-dependent histone demethylases. Molecular modeling revealed that D-2-HG occupies the α-KG binding site and hampers the demethylation reaction of histones. Similarly, D-2-HG competitively inhibits ten-eleven translocation methylcytosine dioxygenase 1 and 2 (TET1 and TET2). TET catalyzes the demethylation reactions involving the conversion of 5-methylcytosine (5-mC) to 5-hydroxymethylcytosine (5-hmC), 5-hmC to 5-formylcytosine (5-fC), and 5-fC to 5-carboxylcytosine (5-caC). The presence of D-2-HG limits the efficacy of cytosine demethylation, which results in the accumulation of 5-mC throughout the genome [15,62]. These findings later confirmed that the acquisition of *IDH1* mutants or high amounts of D-2-HG is sufficient to induce the hypermethylation phenotype identified among patients with *IDH*-mutated gliomas [18]. Epigenetic reprogramming may lead to the oncogenesis of glioma and other malignancies. For example, Flavahan et al. [63] reported that *IDH* mutant gliomas exhibit hypermethylation at cohesin and CCCTC binding factor (CTCF)-binding sites, which compromises the binding of this important insulator protein. CTCF plays a critical role in maintaining the insulation between topological domains and preventing aberrant gene activation. Loss of CTCF binding leads to the constitutive expression of the receptor tyrosine kinase gene *PDGFRA* and promotes gliomagenesis. In addition, the D-2-HG-associated hypermethylation phenotype impedes cellular differentiation by inhibiting lysine-specific demethylase 4C (KDM4C), which may assist in the maintenance of stemness and gliomagenesis [17].

### 3.5. DNA Repair Enzymes

DNA repair is an evolutionarily conserved cellular function, which is commonly exploited by cancer cells against genotoxic therapies, such as radio- and chemo-therapy [64]. DNA repair involves a spectrum of highly sophisticated molecular mechanisms, and employs multiple DNA modification enzymes [65,66]. A growing body of evidence suggests that D-2-HG affects multiple DNA repair pathways. For example, Wang et al. [19] reported that *IDH*-mutant-induced D-2-HG inhibits the α-KG-dependent alkB homolog (*ALKBH*) DNA repair enzymes, which sensitize *IDH* mutant cancers to DNA alkylating agents. In addition, Ohba et al. [67] reported that mutant *IDH1* drives a unique set of transformative events, resulting in increased RAD51-mediated homologous recombination (HR). Moreover, Inoue et al. [20] reported that mutant *IDH1* downregulates the DNA damage sensor ataxia-telangiectasia-mutated (ATM) signaling pathway by altering histone methylation, leading to sensitivity to DNA damaging agents. Another study conducted by Núñez et al. [68] showed that in the context of ATRX loss, *IDH* mutant cancers enhance DNA damage response via the up-regulation of the ATM pathway, suggesting that ATRX deficiency in diffusive astrocytoma may affect DNA repair pathways and sensitivity to therapy. Finally, us and several groups have reported that the poly (ADP-ribose) polymerase-1 (PARP1)-associated DNA repair pathway is compromised extensively in mutant cells because of decreased NAD^+^ availability. Combinations including PARP inhibitors sensitize *IDH* mutant cancers to chemotherapy and radiotherapy [21,22,69]. The seminal research conducted by Sulkowski et al. [23,70] revealed that D-2-HG compromises HR DNA repair by influencing histone methylation and local chromatin signaling. With the substantial expansion in knowledge regarding the D-2-HG-suppressed DNA repair pathway, it is possible to improve the current standard of care for glioma, through the sensitization of cancer cells with molecular targeting approaches against remnant DNA repair pathways.

### 3.6. Metabolic Enzymes

The neomorphic activity of mutant *IDH* completely alters the metabolic flux in the Krebs cycle, and therefore establishes a distinctive pattern in cancer metabolism. Grassian et al. [71] reported that cells expressing mutant *IDH1* show increased oxidative tricarboxylic acid metabolism, along with suppressed reductive glutamine metabolism under hypoxic conditions. Reitman et al. [72] profiled more than 200 metabolites in human oligodendroglioma cells in order to investigate metabolic reprogramming by the *IDH1* mutant enzyme. The researchers discovered that glutamate levels were reduced in cells with *IDH* mutants. The reduction in glutamate levels might indicate that α-KG is replenished by glutaminolysis. Ohka et al. [73] provided additional insights into glutamine catabolism in *IDH*-mutated cells. The researchers reported that glutaminolysis is activated in *IDH* mutant cells. Furthermore, several studies have shown that the inhibition of glutaminases suppresses the growth of *IDH* mutant cancers, which indicates that reduced glutamate and increased dependence on glutaminolysis are key features of *IDH* mutant cancers [29,74].

### 3.7. Anti-Oxidative Pathways

The cellular redox status is maintained by the balance of NADPH/NAPD^+^. The neomorphic activity of the *IDH* mutant enzyme utilizes NADPH as a cofactor, and therefore exhausts the availability of the reductive equivalent for biosynthetic reactions [24,25,26]. Our previous studies have shown that the acquisition of *IDH* mutants is associated with elevated levels of reactive oxygen species (ROS), suggesting a distinctive pattern in redox homeostasis in malignancies with *IDH* mutants. The increased ROS burden was considered harmful to cells, which might lead to catastrophic oxidative damage. Moreover, we discovered that *IDH* mutant cells mobilize multiple anti-oxidative pathways to maintain the fragile redox homeostasis. NRF2-governed anti-oxidative pathways, such as that of de novo glutathione synthesis, play a pivotal role in the manifestation of *IDH*-mutated glioma [25,27,28].

## 4. Clinical Indications Involving the Discovery of *IDH*-Mutated Glioma

### 4.1. Clinical Classification of Gliomas

The 2016 WHO classification of CNS tumors has suggested the use of integrated phenotypic and genotypic characterization, which provides an increased level of objectivity [75]. In particular, *IDH* mutations have become some of the most important parameters in the differential diagnosis of gliomas. For example, diffuse astrocytomas often harbor *IDH* mutations, followed by other mutations such as *TP53* and *ATRX*. Oligodendrogliomas are characterized by *IDH* mutations along with 1p/19q co-deletion (potentially along with *CIC* and *FUBP1* mutations). The *IDH* mutation status is also useful for the differential diagnosis of primary and secondary GBMs [75,76,77]. Moreover, as *IDH* mutations frequently induce genome-wide DNA and histone hypermethylation, the introduction of methylation profiling allows for further improving the accuracy of glioma classification. Recently, Jaunmuktane et al. [78] demonstrated a diagnostic algorithm that integrated histology, molecular signature, and methylation array, and improved the diagnostic approach. Thus, the *IDH* mutation status is of great value in glioma classification and the selection of appropriate therapeutic strategies.

### 4.2. Radiology—D-2-HG Imaging

D-2-HG is a novel metabolite that accumulates in extremely high levels in glioma cells, but is absent in normal brain cells. The drastic contrast in cellular D-2-HG levels suggests that this oncometabolite could be an ideal biomarker for clinical monitoring and diagnosis among patients with *IDH*-mutated cancers [79]. Several hallmark studies have developed noninvasive radiologic methods for the detection of D-2-HG, such as magnetic resonance spectroscopy (MRS). In *IDH* mutant gliomas, D-2-HG accumulates to sufficient levels as a brain metabolite, which renders its visibility on MRS. These levels are 2–3 orders of magnitude higher than those found in the adjacent normal brain tissues [79]. Andronesi et al. [80] reported that D-2-HG was detected unambiguously in mutant *IDH1* glioma in vivo using 2D correlation spectroscopy (COSY) and J-difference spectroscopy. Several other studies have also reported that D-2-HG is detected among glioma patients or in animal models using the short echo times (TEs) method [81,82,83,84]. On the other hand, D-2-HG levels were detected in glioma patients using long TE methods and J-difference spectroscopy with 100% sensitivity [85,86]. The application of long TE methods in D-2-HG detection has been confirmed in several subsequent reports, with increased sensitivity and specificity [87,88]. Overall, the noninvasive detection of D-2-HG has been proven to be a valuable diagnostic and prognostic biomarker. D-2-HG imaging provides a useful approach to the clinical management of patients with *IDH*-mutated glioma. Fluctuations in D-2-HG levels may provide crucial longitudinal data for the determination of disease progression and therapy response [79].

### 4.3. Disease Outcomes—Prolonged Survival

**Figure 3:**
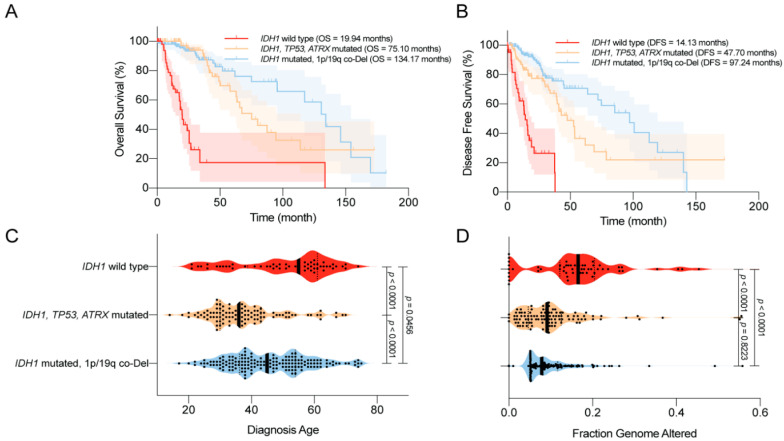
Clinical features of the World Health Organization (WHO) grade II/III *IDH*-mutated glioma. (**A**) Overall survival (OS) of glioma patients according to *IDH1* status. *IDH1* mutations are associated with prolonged OS. (**B**) Disease-free survival (DFS) of glioma patients according to *IDH1* status. *IDH1* mutations are associated with prolonged DFS. (**C**) Age at diagnosis among glioma patients according to *IDH1* status. *IDH1* mutations are associated with a younger age at diagnosis. (**D**) The distribution of genome alterations in glioma according to *IDH1* status. *IDH1* mutations are associated with fewer genome alterations. The data are visualized in cBioPortal [46,47].

In 2008, Parsons et al. [1] first reported that mutations in *IDH1* occurred in most patients with secondary GBM, and were associated with better overall survival (OS). Similar trends were reported in numerous studies using various datasets [42,89,90,91,92,93]. For example, using a large clinical dataset, Yan et al. [2] reported that GBM patients harboring *IDH1* or *IDH2* mutations tend to have a prolonged median OS compared with patients with *IDH* wild-type GBM. Similar findings were also observed among patients with anaplastic astrocytoma. The median OS was 65 months for patients with *IDH* mutant disease, compared with 20 months for those with *IDH* wild-type disease. Moreover, the progression-free survival (PFS) was also improved among GBM patients with *IDH* mutations compared with their counterparts [89]. Secondary genetic alterations, such as *TP53*/*ATRX* mutations and 1p/19q co-deletion, predispose patients with *IDH*-mutated gliomas to slightly different OS and disease-free survival (DFS; Figure 3A,B). Several studies have reported that *IDH* mutations are associated with younger age at diagnosis and limited genome alterations among patients with WHO grade II/III gliomas and GBMs, which may bias the disease outcome (Figure 3C,D) [1,2,94,95]. However, in a multivariate analysis, Sanson et al. [89] showed that the *IDH* mutation status is an independent predictor of favorable outcomes among glioma patients.

### 4.4. Complications—Epilepsy and Secondary GBM

Epileptic seizure is one of the most common complications among patients with glioma, particularly those with LGGs (up to 90%) [96,97,98,99]. Severe seizures impair the quality of life and neurocognition function among glioma patients [100]. Considering the high incidence of *IDH* mutations in LGG, it is likely that the epileptic changes are relevant to the unique patterns in the tumor microenvironment, which is associated with *IDH* mutants. Numerous studies have indicated that mutations in *IDH* are associated with a high prevalence of epilepsy [101,102,103,104,105]. For example, Chen et al. [104] showed that *IDH* mutations are independently correlated with seizures, regardless of WHO grade. A recent study suggested that D-2-HG overproduction in the tumor microenvironment plays a major role in glioma-related epilepsy. D-2-HG is structurally similar to glutamate, which is the predominant excitatory neurotransmitter in the CNS. Thus, D-2-HG may act as an analog of glutamate, which leads to the abnormal firing of neurons through activating N-Methyl-d-aspartic acid (NMDA) receptors, and hence epileptic changes. Treating cultured rat cortical neurons with exogenous D-2-HG resulted in an elevated firing rate [104]. By mimicking the activity of glutamate, the increased level of D-2-HG mediates the abnormal neuronal activity and leads to glioma-related epilepsy [80,106,107]. However, three millimolar D-2-HG induced an elevated burst frequency in the neuronal network in vitro [104], whereas this dose is over 30 times higher than the glutamate concentration for excitotoxicity [108]. More effort is urged in order to elucidate the detailed molecular mechanism of the epileptic changes in *IDH*-mutated glioma. Because of the association between *IDH* mutations and seizures, therapies that target mutant *IDH*, such as mutant *IDH* inhibitors, could diminish D-2-HG production and potentially reduce epileptic seizures [109].

### 4.5. Sensitivity to Radiotherapy and Chemotherapy

Clinical data have shown that *IDH* mutant gliomas tend to exhibit a better disease outcome compared with wild-type *IDH* tumors. Several studies have explained that the favorable prognosis of *IDH* mutant gliomas is due to their increased sensitivity to radiotherapy and chemotherapy [110,111]. *IDH* mutant gliomas likely harbor defects in multiple DNA repair pathways, which render them vulnerable to radiotherapy- or chemotherapy-induced DNA damage [19,22]. These findings indicate that *IDH* mutation could serve as an important predictive factor for treatment response among glioma patients. For example, Houillier et al. [111] reported that *IDH1* mutation is an independent predictor of temozolomide response among LGG patients. *IDH1* mutations combined with 1p/19q co-deletion further improved the treatment response. Hartman et al. [112] also reported that *IDH1* status is an important predictor of disease-free survival (DFS) and OS among patients undergoing adjuvant therapy. In another study conducted by van de Bent et al. [113], no correlation was found between *IDH1* mutations and disease outcome in response to procarbazine (Matulane), lomustine (CCNU), and vincristine (Oncovin) chemotherapy.

## 5. Novel Molecular Targeting for *IDH*-Mutated Glioma

### 5.1. IDH Mutant Inhibitors

Because of the critical roles played by *IDH* mutations in the malignant transformation of glioma, targeting the neomorphic activity of *IDH* mutants has been heavily proposed as a direct therapeutic approach. In the past decade, several attempts have been made to develop small molecular compounds that directly inhibit mutant *IDH* enzymes. In 2012, the first-in-class mutant *IDH* inhibitor was discovered, which showed a specific and potent inhibitory effect on D-2-HG production in *IDH* mutant U87 cells and xenograft models [114]. Later, Rohle et al. [109] reported a novel synthetic inhibitor of *IDH* mutant, AGI-5198, which blocked D-2-HG production and subsequently reversed the malignant transformation effect of *IDH* mutations. Besides glioma, the inhibition of mutant *IDH* promotes differentiation in leukemia harboring *IDH* mutations [6]. With the promising findings regarding AGI-5198, second-generation mutant *IDH* inhibitors are under development and are undergoing evaluation in clinical studies. For example, ivosidenib (AG-120) and vorasidenib (AG-881) have been tested in AML and glioma with *IDH* mutations [115,116,117,118]. In a recent phase I clinical study with ivosidenib in *IDH1*-mutated advanced glioma conducted by Mellinghoff et al. [119], the mutant *IDH* inhibitor appeared to be well-tolerated throughout the experiment, which paved the way for subsequent clinical studies to evaluate its therapeutic efficacy. Although the *IDH* mutant enzyme inhibitors suppress malignancy, several studies have suggested that this inhibitor reduces D-2-HG production and relieves the burden on the DNA repair pathway, resulting in chemoresistance to other therapies, such as PARP inhibitors [23,120]. More effort is urged to explore the strategy of combining *IDH* mutant inhibitors with other glioma therapies in order to improve the clinical outcome.

### 5.2. Targeting Hypermethylation Phenotype

Genome-wide DNA and histone hypermethylation is a unique signature in *IDH*-mutated glioma, which is closely related to gliomagenesis by promoting oncogene expression and inhibiting tumor suppressors [63]. This rectification of the epigenetic shift could be a reasonable strategy for halting D-2-HG-driven oncogenesis and the malignant phenotype. DNA-demethylating agents such as 5-azacytidine or 5-aza-2′-deoxycytidine (decitabine) irreversibly bind to DNA methyltransferases (DNMTs) and inhibit the process of DNA methylation. The D-2-HG-induced hypermethylation phenotype was reversed by demethylating compounds, and cell proliferation was suppressed in vitro and in vivo [121,122,123]. Several clinical trials are evaluating the therapeutic effects of 5-azacytidine among patients with recurrent gliomas with *IDH* mutations (NCT03666559 and NCT03684811). On the other hand, inhibitors targeting histone methyltransferases inhibitors are also being investigated for *IDH*-mutated gliomas, as an alternative strategy to rectify the D-2-HG-associated hypermethylation phenotype. It is reported that H3K9 methyltransferase G9a is correlated to the development and progression of glioma, and its inhibitor BIX-01294 showed repressive effects on gliomas cells [124].

### 5.3. Targeting DNA Repair Pathways

As previously mentioned, *IDH* mutant gliomas exhibit defects in multiple DNA repair pathways. High levels of D-2-HG inhibit the activity of DNA oxidative demethylases, such as AlκB homolog 2/3 (*ALKBH2*/*3*) [19]. Several seminal studies have also indicated that D-2-HG compromises HR DNA repair, establishing a “BRCAness” in this type of malignancy [23,125]. In addition, *IDH* mutation-associated G-CIMP resulted in the methylation of the promoter region of O-6-methylguanine-DNA methyltransferase (MGMT), which reduced MGMT expression and led to increased sensitivity to alkylating agents [90,126,127]. Our recent study indicated that *IDH* mutations led to defects in NAD metabolism, which compromised PARP-associated HR, as PARP repairs DNA damage in an NAD^+^ dependent manner [22,69]. With the identification of the DNA repair deficiency in *IDH*-mutated glioma, numerous studies have attempted to evaluate DNA repair inhibitors, which may serve as a potential sensitization strategy. Several other groups and as well as ours reported that a combination of PARP inhibitors, such as olaparib, with temozolomide or radiotherapy, led to synergistic lethality in *IDH* mutant glioma cells [21,22,23]. Several phase I/II clinical trials are currently recruiting patients to investigate the therapeutic effect of the PARP inhibitors, pamiparib (BGB-290) or olaparib, combined with temozolomide in *IDH* mutant gliomas (NCT03914742, NCT03749187, and NCT03212274).

### 5.4. Targeting Anti-Oxidative Pathways

Redox homeostasis has been reported to be greatly impacted by *IDH* mutations, highlighted by profoundly elevated levels of oxidative stress [24,25,26,27,28]. As a result, ROS scavenging pathways are widely mobilized in the context of *IDH* mutation, so as to maintain cellular metabolism, thereby supporting cellular growth and survival. These findings suggest that the antioxidant pathway plays an essential role in *IDH*-mutated glioma. Targeting anti-oxidative pathways may be more effective in glioma with *IDH* mutations. Our recent study showed that NRF2-governed anti-oxidative pathways, such as that regarding de novo glutathione synthesis, were widespread in *IDH* mutant gliomas. The blockade of NRF2 using natural compound inhibitors, brusatol, or triptolide significantly increased oxidative damage and subsequently suppressed the growth of *IDH* mutant xenografts with prolonged OS [25,27,28,128]. The concept of targeting redox homeostasis in *IDH* mutant cancers has shown a potential therapeutic value. The development of pharmacological grade NRF2 inhibitors is needed urgently for potential clinical translation.

### 5.5. Targeting Metabolic Reprogramming

D-2-HG is a metabolite that is absent in normal cells. The production of large quantities of D-2-HG inevitably depletes a substantial amount of carbohydrate from the Krebs cycle. Several hallmark studies have demonstrated the presence of depleted metabolic pathways in *IDH*-mutated cells. For example, glutamate metabolism is greatly altered in *IDH* mutant glioma, as mentioned before. The glutamate level is significantly lower in *IDH* mutant cancers, which leads to an increased dependence on glutaminolysis to compensate for the metabolism [29,74,129,130]. Several studies have reported that a blockade of glutaminase activity results in the suppression of *IDH* mutant glioma and AML. Seltzer et al. and Emadi et al. [74,129] reported that bis-2-(5-phenylacetamido-1,2,4-thiadiazol-2-yl) ethyl sulfide (BPTES), an inhibitor of glutaminase, selectively suppresses tumor growth in *IDH* mutant glioma and AML by targeting the fragile glutamine metabolism. Another glutaminase inhibitor (CB-839) was also reported to induce selective radio-sensitivity in *IDH* mutant cancers [29] and terminal differentiation in *IDH* mutant AML [130]. An ongoing phase I clinical trial is investigating the side effects and the best dose of CB-839, in combination with radiation therapy and temozolomide, for treating *IDH*-mutated diffuse or anaplastic astrocytoma (NCT03528642). In addition, *IDH* mutations lead to the depletion of NAD^+^ because of the increased methylation of the promoter region of *NAPRT1*, the rate-limiting enzyme in NAD^+^ biosynthesis, and suppression of the expression of NAPRT1. This renders the *IDH* mutant glioma vulnerable to inhibition through the nicotinamide phosphoribosyltransferase (NAMPT) catalyzed NAD^+^ salvage pathway [131]. Moreover, Tateishi et al. [132] showed that NAMPT inhibitors further sensitized *IDH* mutant cancer cells to alkylating agents, such as temozolomide, as PARP activation consumes NAD^+^ during the base excision repair of chemotherapy-induced DNA damage. With the substantially exhausted metabolic pathways, distinctive metabolic vulnerabilities are established in *IDH*-mutated malignancies. Effectively targeting these metabolic pathways may induce selective cytotoxicity to cancer cells, but a lesser extent than that occurs in normal somatic cells with an intact metabolic network.

### 5.6. Immunotherapies

The accumulated pieces of evidence have indicated that *IDH* mutant cancers exhibit an immunosuppressive tumor microenvironment [133,134]. Bunse et al. [133] reported that the D-2-HG produced by mutant *IDH* is taken up by T cells and suppresses T cell activity. Moreover, Kohanbash et al. [134] reported that *IDH* mutations inhibit STAT1 expression, and subsequently attenuate CD8^+^ T cell accumulation in gliomas. This evidence indicates that effective immunotherapy in *IDH* mutant cancers could be challenging. However, numerous studies have suggested different approaches to overcoming *IDH* mutant related immunosuppression, typically by *IDH* mutant-specific peptide vaccine [135] or by using immune checkpoint inhibitors [133,136]. For example, cancer cells with the *IDH1* R132H variant present a tumor-specific CD4^+^ T cell neoepitope. Peptide vaccination targeting the *IDH1* R132H mutation results in an effective anti-tumor immune response, and suppresses the growth of pre-established *IDH1* R132H-mutated tumors [135]. Moreover, the inhibition of the mutant *IDH* neomorphic enzymatic activity improves the anti-tumor immunity of the *IDH1*-specific vaccine [134]. Vaccine-based immunotherapies have been developed based on the neoantigen targets in gliomas. For example, EGFR variant III (EGFRvIII) is the most common mutation in *IDH1* wild-type GBMs. A peptide vaccine, rindopepimut (CDX-110), was developed to target this mutation. A recent phase III clinical trial showed that, although rindopepimut induced a decent humoral immune response, no significant survival benefit was observed [137,138,139]. Several *IDH1* peptide vaccines are currently in early phase clinical trials (e.g., NCT02454634, NCT03893903, and NCT02193347), which should provide critical information regarding the safety and efficacy of this approach. Recently, applications of immune checkpoint inhibitors have been brought into the spotlight of clinical investigations [136]. However, in *IDH* mutant cancers, the immunosuppressive microenvironment might limit the therapeutic efficacy of immune checkpoint inhibitors. Bunse et al. reported that the inhibition of mutant *IDH* resulted in an enhanced anti-tumor effect of anti-PD-1 treatment [133].

## 6. Conclusions—Current Knowledge and Opportunities for the Future

In summary, significant amounts of effort and progress have been made over the past decade to understand the biology of *IDH* mutations in glioma. A growing body of evidence has shown a correlation between *IDH* mutation and malignant transformation by altering cellular epigenetics, metabolism, DNA repair pathways, and redox homeostasis. This provides potential opportunities for targeting these pathways as therapeutic approaches to *IDH* mutant cancers, for example, targeting hypermethylation phenotype using epigenetic modulators [63,121,122]; targeting essential metabolic pathways, such as the NAD de novo synthesis pathway [131] or glutaminolysis [29,140]; targeting compromised DNA repair pathways in *IDH* mutant cancers [22,23,69,141]; and targeting redox regulators, such as NRF2 [25,27,28,128]. Moreover, with the increased knowledge of the molecular mechanism, targeting *IDH* mutations is suggested as a therapeutic approach to cancers bearing these mutations. Specific small molecular inhibitors of mutant *IDH* have been developed to inhibit the *IDH* mutant neomorphic activity [109]. Although several studies have reported some limitations to their application [23,142,143], mutant *IDH* inhibitors have still shown promising therapeutic benefits in numerous preclinical and clinical studies [116,144,145]. The recent development of glioma mouse models has provided generous insights regarding glioma biology and therapeutics [146]. An earlier study by Bardella et al. [32] showed that the mice with a *IDH1* R132H expression developed hydrocephalus and grossly dilated lateral ventricles; however, only precursor lesions were observed. Several recent advances in *IDH1* mutant mouse models have been developed, which are discussed in our recent review [53], along with increased evidence reflecting the potential value of targeting mutant *IDH* in cancer treatment. Thus, more efforts are needed to elucidate the role of *IDH* mutation in tumorigenesis and clinical translation.
